# Enhancing Polylactic Acid (PLA) Performance: A Review of Additives in Fused Deposition Modelling (FDM) Filaments

**DOI:** 10.3390/polym17020191

**Published:** 2025-01-14

**Authors:** Ioan Plamadiala, Catalin Croitoru, Mihai Alin Pop, Ionut Claudiu Roata

**Affiliations:** 1 Materials Engineering and Welding Department, Transilvania University of Brasov, 500036 Brasov, Romania; ioan.plamadiala@unitbv.ro (I.P.); ionut.roata@unitbv.ro (I.C.R.); 2 Department of Materials Science, Transilvania University of Brasov, 500036 Brasov, Romania; mihai.pop@unitbv.ro

**Keywords:** PLA, mechanical properties, additives, carbon fiber, glass fiber, additive manufacturing, graphene, CNT

## Abstract

This review explores the impact of various additives on the mechanical properties of polylactic acid (PLA) filaments used in Fused Deposition Modeling (FDM) 3D printing. While PLA is favored for its biodegradability and ease of use, its inherent limitations in strength and heat resistance necessitate enhancements through additives. The impact of natural and synthetic fibers, inorganic particles, and nanomaterials on the mechanical properties, printability, and overall functionality of PLA composites was examined, indicating that fiber reinforcements, such as carbon and glass fibers, significantly enhance tensile strength and stiffness, while natural fibers contribute to sustainability but may compromise mechanical stability. Additionally, the inclusion of inorganic particulate fillers like calcium carbonate improves dimensional stability and printability, although larger particles can lead to agglomeration issues. The study highlights the potential for improved performance in specific applications while acknowledging the need for further investigation into optimal formulations and processing conditions.

## 1. Introduction

The increasing demand for functional prototypes and end-use parts has driven the rapid development of Additive Manufacturing (AM) technologies, with Fused Deposition Modeling (FDM) or Fused Filament Fabrication (FFF) being one of the most popular methods. FDM relies on filaments, which are fed through a heated extruder and deposited layer-by-layer to create 3D objects. Choosing the right filament material is crucial for successful printing and achieving the desired final product properties. Over recent years, there has been growing interest in biopolymers as sustainable FDM alternatives to petroleum-based materials [1]. 

Among biopolymers, polylactic acid (PLA) stands out for its widespread production from renewable resources (240,000 tons annually) and its biodegradability and biocompatibility [2]. 

**Figure 1:**
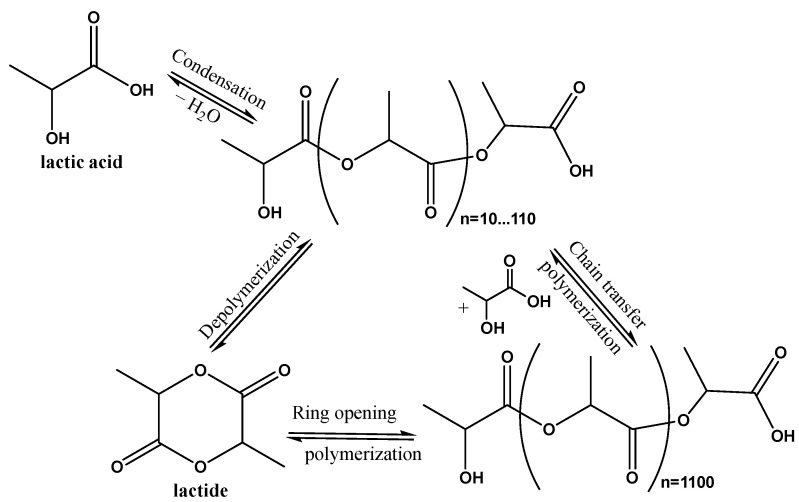
Main reaction pathways for polylactic acid (PLA) synthesis.

These qualities make PLA attractive for various applications, including commodity goods, medical devices, and FDM 3D printing [2]. PLA can be synthesized through several pathways, including condensation of lactic acid or ring-opening polymerization of lactide (its cyclic dimer) in the presence of tin octanoate or zinc stearate catalysts (Figure 1) [3].

**Figure 2:**
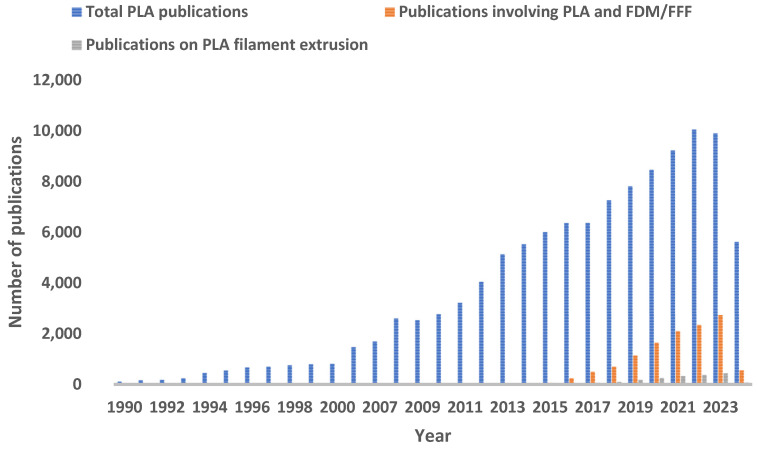
Number of publications involving PLA indexed in the Clarivate ISI Web of Science database (accessed on 2 January 2025).

There is a noticeable upward trend in the total number of publications on PLA, as extracted from the Clarivate ISI Web of Science database (Figure 2), indicating increasing interest and research activity in this biodegradable polymer. Although the number of publications specifically involving PLA and 3D printing/FDM is less than the total PLA publications, it also shows a rising trend. This suggests that the use of PLA in 3D printing is gaining traction within the research community, especially since 2015. The growing number of publications could reflect advancements in PLA applications, improvements in 3D printing technology, and a broader recognition of the importance of sustainable materials. The same increasing trend could be seen in the number of studies involving 3D printing with custom-made (non-commercial) PLA filaments.

**Figure 3:**
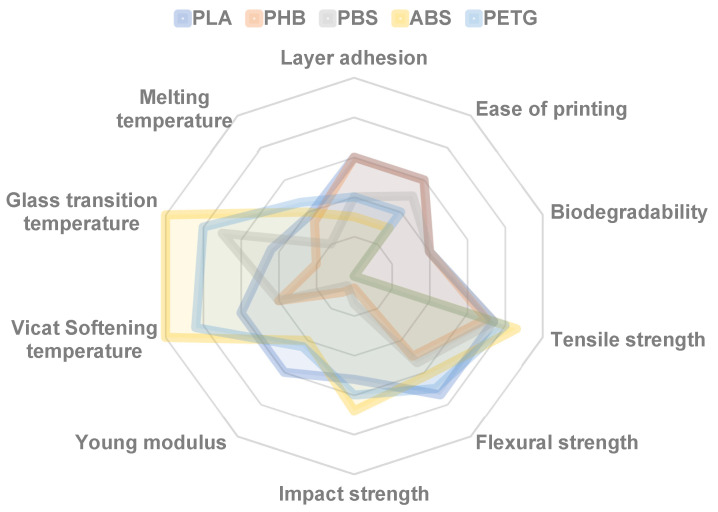
Main properties of commonly used fused deposition modeling (FDM) polymers in pure form (relative comparison) based on data from [6].

PLA’s popularity in FDM stems from several advantages. First, its low melting temperature (150–180 °C) allows for easy printing and good layer adhesion, enabling intricate designs [4]. However, PLA also has limitations for FDM applications compared to other polymers, including those from the polyester class, such as polyhydroxybutyrate (PHB), polybutylene succinate (PBS), polyethylene terephthalate glycol (PETG) or poly(acrylonitrile-co-butadiene-co-styrene) (ABS), as outlined in Figure 3 [5].

Compared to ABS or PETG, PLA has lower strength and heat resistance. Its heat deflection temperature (HDT) is 53–56 °C, meaning PLA parts can deform under elevated temperatures [5]. Additionally, PLA exhibits good tensile strength but lacks flexibility, making it unsuitable for load-bearing applications. Furthermore, PLA’s high thermal expansion coefficient can cause warping and deformation during printing. 

Additionally, PLA is not as strong or durable as other FDM materials to organic solvents (e.g., acetone, ethyl acetate, toluene, dichloromethane, aliphatic ketones), as the printed parts either dissolve or delaminate when exposed to these compounds. Also, more challenging alkaline/acidic conditions and humid environments can pose a problem, which may affect the longevity and performance of printed objects. 

PLA’s inherent limitations can be addressed through the incorporation of various fillers and additives into filaments. These can improve mechanical properties, thermal resistance, and printability.

While PLA is often praised for being an eco-friendly material in 3D printing, a deeper examination reveals a more nuanced picture. Typical PLA composite filaments for FDM applications go beyond just the reinforcing materials (like fibers or powders). They also include a surprising array of additional ingredients [7]. These additives, although necessary to achieve desired properties, can raise concerns about the overall environmental impact and efficiency [8,9,10,11,12]. The most important additives used in PLA filaments are the following:

- Coupling Agents: These improve the adhesion between the reinforcing material and the PLA matrix. Examples include silanes and maleic anhydride grafted polymers, with typical loading ratios of 0.5–3 wt. %
- Impact Modifiers: These additives enhance the toughness and impact resistance of the composite. They can be elastomeric polymers (e.g., thermoplastic polyurethane, TPU), core–shell particles, or other toughening agents, usually added in 5–15 wt. % ratios.
- Processing Aids: Lubricants (fatty acids or waxes at 0.5–3 wt. % loading) and plasticizers (e.g., poly (ethylene glycol) (PEG) at typical loadings of 5–10 wt. %) improve the flow and printability of the filament during printing.
- Nucleating Agents and Antioxidants: These additives promote crystallization, improve thermal properties, and prevent degradation of the PLA during processing and use. Examples include talcum powder, calcium carbonate (1–5 wt. %), phenolic antioxidants, and UV absorbers (0.1–3%).
- Processing, since crystallinity results in poor dimensional stability due to shrinkage and
- Warpage in FDM processes.
- Flame Retardants: While these enhance safety, they often require high loading percentages, impacting printability and potentially adding weight (e.g., aluminum trihydroxide at 10–13 wt. %).
- Colorants: These add desired colors to the filament and printed parts without significantly affecting mechanical properties.

Despite the advancements, challenges remain in using additives with PLA for FDM printing. These challenges include compatibility issues between the additives, the filler material, and the PLA itself. Additionally, optimizing printing parameters and ensuring scalability of the manufacturing process are ongoing areas of research [3]. While these additives can improve mechanical performance, they may also lead to lower adhesion between layers compared to pure PLA. This is because faster solidification and larger voids can occur during printing with additives [13].

Future research should focus on overcoming these compatibility challenges and exploring the potential of PLA formulations with natural components for FDM printing [3]. A key area is achieving better dispersion of additives within the PLA matrix, which is crucial for optimal mechanical properties and avoiding defects in printed parts [3]. It is also important to consider the cost-effectiveness of additives, as some can be expensive and hinder biodegradability or the commercial viability of PLA filaments for certain applications [14].

Despite challenges like uneven printing, clogging, and filler–matrix distribution, natural fiber-reinforced PLA composites hold promise in addressing these issues [15], making the 3D-printed materials suitable for a wider range of applications [5,16]. For instance, adding cellulose-based fibers up to 30 wt. % can significantly increase PLA’s stiffness and impact strength without impacting the density and environmental footprint of the material [17,18].

Researchers are pushing the boundaries of PLA’s performance by exploring a wider range of fillers and reinforcements beyond natural fibers. This includes incorporating various synthetic fibers and particulate materials like metallic particles, ceramics, polymers or biomass-derived particles (e.g., sawdust). Metallic particles can enhance thermal conductivity, wear resistance, and electrical conductivity of PLA composites [19,20]. Certain iron or cobalt fillers can impart new properties in PLA, such as magnetic properties, which could be useful in sensor-based applications. However, achieving homogeneous dispersion and mitigating potential increases in weight and brittleness depending on the metal used are ongoing challenges. Similarly, ceramics can improve wear resistance, flame retardancy, and stiffness, but ensuring good dispersion and avoiding negative effects on printability and mechanical properties (the stress concentrator effect) require further research [21]. Both polymeric additives and biomass particles can significantly enhance the properties of PLA, making it more suitable for functional applications. Polymeric additives can improve toughness, and could strengthen the adhesion between PLA and reinforcing fibers [22]. 

Biomass particles, such as wood or micronized plant materials, can significantly increase strength, making PLA composites suitable for demanding applications [23,24].

Nanomaterial fillers offer even more exciting possibilities due to their high surface area-to-volume ratio. These materials have the potential to significantly improve properties like strength, flame retardancy, and barrier properties within PLA composites [25,26]. However, safe handling and proper integration of these materials within the PLA matrix necessitate further investigation. 

This paper aims to provide an overview of the potential of PLA filaments filled with various reinforcing agents for FDM/FFF printing. The focus will be on how the incorporation of natural and synthetic fibers (like glass and carbon fibers), inorganic particles (such as calcium carbonate), organic biomass particles, nanomaterials or polymers can enhance the properties of PLA filaments. The advantages and disadvantages of these fillers/additives are discussed, focusing on their impact on mechanical strength, heat resistance, printability, and overall functionality of the printed parts. By examining the current state of research on filled PLA filaments, this paper aims to emphasize future developments in this field and promote the use of PLA for a wider range of FDM/FFF printing applications.

## 2. Reinforcing Agents for PLA FDM/FFF Applications

### 2.1. Fibers

In the realm of FDM 3D printing, PLA filament manufacturers have embraced the use of fiber reinforcements to elevate the material’s mechanical properties and, in some cases, its environmental footprint. These reinforcements come in two main categories: synthetic and natural fibers.

Synthetic fibers, including carbon fiber, glass fiber, and Kevlar, are popular choices for reinforcing PLA due to their exceptional strength and stiffness. This translates to significantly enhanced mechanical properties in the resulting composites. They typically exhibit improvements in tensile strength, modulus, and impact resistance. For example, carbon fiber reinforcement can lead to a remarkable 150% increase in tensile strength, while glass fiber can boost the modulus by a range of 50–100%. Additionally, these synthetic fibers enhance the thermal stability and heat deflection temperature of PLA. Notably, carbon fiber can significantly improve thermal conductivity, making the composite suitable for high-temperature applications [27].

However, incorporating synthetic fibers presents a challenge in the filament extrusion process. Specialized equipment and techniques become necessary to ensure uniform fiber dispersion and prevent clogging during FDM printing.

Natural fibers like kenaf, hemp, flax, and bamboo are gaining traction as sustainable alternatives to synthetic fibers. Their biodegradability, renewability, and often lower cost align well with the eco-friendly nature of PLA. While not as dramatic as synthetic fibers, natural fibers enhance the tensile strength and modulus of PLA composites. Kenaf and hemp fibers, for instance, can increase tensile strength by 20–50%, with flax and bamboo offering similar improvements.

The sustainability benefits of natural fibers are undeniable. They are renewable, biodegradable, and have a lower environmental impact compared to their synthetic counterparts. Additionally, natural fibers are less expensive, making composites more affordable. They also offer a weight advantage, which can be crucial in applications where weight reduction is a priority.

However, natural fibers come with their own set of challenges. Their hygroscopic nature, meaning they tend to absorb moisture, can affect the mechanical properties and dimensional stability of the composites. This can be a significant drawback in applications where moisture resistance is critical. Furthermore, the properties of natural fibers can vary depending on factors like source, growing conditions, and processing methods. This variability can translate to inconsistencies in the performance of the final composites. Like synthetic fibers, incorporating natural fibers into PLA filaments can pose challenges during the extrusion and FDM printing processes, requiring careful attention to ensure uniform dispersion and prevent clogging. Some strategies that mitigate clogging are as follows:

- Using a large nozzle: Fibers can be larger than standard PLA fillers, so using a larger nozzle (e.g., 0.6 mm or 0.8 mm) can help prevent clogging.
- Printing at a higher temperature: Printing at a slightly higher temperature than standard PLA (around 200–220 °C) can help the fibers flow more easily through the nozzle.
- Reducing retraction: Minimizing retraction distance and speed can help prevent the filament from being pulled back into the nozzle, which can cause clogging.
- Adjusting printing speed: Printing at a slower speed can give the filament more time to melt and flow through the nozzle, reducing the risk of clogging.
- Drying the filament: Especially natural fibers can absorb moisture from the air, which can cause inconsistent extrusion and clogging.

#### 2.1.1. Carbon Fiber

Carbon fiber-reinforced PLA (CFR-PLA) is one of the most popular composites. There are two main types of carbon fiber reinforcement: chopped and continuous. Chopped fibers, the most common option, are short lengths dispersed within the PLA, offering easier printing but with a lower reinforcement potential. Continuous fibers, embedded in a polymer sheath, provide the highest level of strength and stiffness but require specialized printers due to potential clogging and higher processing temperatures. The choice between these options depends on your priorities [28]. Chopped fibers are ideal for affordability and moderate improvements, while continuous fibers offer the best performance but necessitate a more advanced printing setup.

Typical fiber loadings range from 5% to 20% by weight. For instance, a study reported that 15% carbon fiber loading in PLA increased the tensile strength by over 100% compared to pure PLA [29]. Other studies conversely report reduced tensile strength but increased Young’s modulus and elongation at break, for example, from 1.04 GPa and 6.26%, to 1.26 GPa and 7.81%, respectively [30,31]. The main weakness in these PLA-CF composites lies in the fiber–matrix interface, with fiber pull-out being the primary failure mode due to interface destruction. This effect is more pronounced at higher fiber loads reported to the PLA matrix [32].

The mechanical properties of 3D-printed carbon fiber PLA composite are significantly influenced by the FDM process parameters. The following parameters have been found to have a significant impact on the mechanical properties of CFR-PLA, as referenced from the literature [32,33,34]:

- Nozzle geometry: Square nozzle geometry increases tensile strength and reduces void geometry (with up to 7%) in carbon nanofiber-reinforced polylactic acid composites during FDM printing [35]. Also, this geometry enhances the fracture toughness with less inter-bead voids and larger bonded areas in carbon fiber-reinforced PLA composites [36].
- Printing Layer Height and print orientation: The layer height has a significant effect on the mechanical properties of CFR-PLA. A lower layer height (0.1–0.2 mm) can result in improved tensile strength and Young’s modulus, while a higher layer height (0.4–0.6 mm) can lead to a decrease in mechanical properties [37]. A study mentions that a print orientation of 45° presents superior mechanical strengths [38]. Optimal process parameters for enhancing the impact strength of carbon fiber-reinforced PLA during FDM printing include layer thickness of 0.04 mm and shell thickness of 1.2 mm [39].
- Extrusion Width: The extrusion width has a significant impact on the mechanical properties of CFR-PLA. A wider extrusion width (0.4–0.6 mm) can result in improved tensile strength and Young’s modulus, while a narrower extrusion width (0.1–0.2 mm) can lead to a decrease in mechanical properties [32].
- Printing Temperature: The printing temperature has a significant effect on the mechanical properties of CFR-PLA. A higher printing temperature (230–250 °C) can result in improved tensile strength and Young’s modulus, while a lower printing temperature (200–220 °C) can lead to a decrease in mechanical properties and lower dispersability of the fiber in the polymer melt [40]. Several studies indicate that the maximum tensile properties for carbon fiber-reinforced PLA are attained at a nozzle temperature of 230 °C [41,42]. Higher nozzle temperatures in the FDM process increase the crystallinity of printed PLA, promoting its nucleation, thus enhancing mechanical properties and rheological properties [13].
- Printing Speed: The printing speed has a significant impact on the mechanical properties of CFR-PLA. A slower printing speed (10–20 mm/s) can result in improved tensile strength and Young’s modulus, while a faster printing speed (50–100 mm/s) can lead to a decrease in mechanical properties [38].
- Infill Percentage: Increasing the infill ratio in FDM printing increases the mechanical properties of both polylactic acid and carbon fiber-reinforced polylactic acid, with a maximum tensile strength of 35.65 MPa for PLA [43]. A higher infill percentage (50–70%) can result in improved tensile strength and Young’s modulus, while a lower infill percentage (20–30%) can lead to a decrease in mechanical properties [44].

#### 2.1.2. Glass Fiber

The addition of glass fibers (GFs) to 3D-printed PLA composites significantly enhances their mechanical and thermal properties. Optimal fiber content and innovative printing methods can further improve these properties, making GF-reinforced PLA composites suitable for a wide range of engineering applications. However, considerations such as moisture sensitivity and layer adhesion need to be addressed to fully leverage the benefits of glass fiber reinforcement.

- Mechanical Strength Enhancement: The addition of glass fibers significantly improves the tensile and flexural strength of PLA composites. For instance, composites with 30% glass fibers show superior mechanical properties compared to neat PLA. Continuous glass fiber reinforcement in PLA composites can achieve high flexural strength and modulus, reaching up to 312 MPa and 21.5 GPa, respectively [45,46,47]. The mechanical properties of PLA composites are optimized at specific glass fiber contents. For example, tensile strength and flexural strength are maximized at 20–30% glass fiber content, while impact strength peaks at 45% [45,48].
- Thermal Stability: GF addition enhances the thermal stability of PLA composites. Composites with 30% glass fibers exhibit excellent thermal stability, making them suitable for applications where thermal resistance is crucial. The heat deflection temperature and linear-expansion coefficient are significantly improved with the addition of short glass fibers [49]. Glass fiber-reinforced polylactic acid composites with isothermal heat treatment significantly improve mechanical properties and thermal stability, enabling their use in applications like automotive, aerospace, and electronics [50]. A study reported that PLA-GF composite filaments are thermally stable until 311 °C [51].
- Layer Adhesion and Printability: The incorporation of glass fibers can improve the printability and dimensional stability of PLA composites, although it may also lead to faster solidification and larger voids, which can affect layer adhesion [13,46].
- Hygromechanical Properties: Glass fiber-reinforced PLA composites exhibit anisotropic behavior, with better performance in the longitudinal direction compared to the transverse direction. Moisture uptake can lead to a reduction in mechanical properties, particularly in the transverse direction [52].
- Chemical and structural stability: While glass fiber reinforcement in PLA (10–30% loading) significantly improves its strength and stiffness for demanding 3D printing applications, a study revealed interesting effects on weatherability. Compared to neat PLA, glass fiber-reinforced PLA (GF-PLA) showed improved mechanical properties even after exposure to sunlight and moisture (weathering). This is because the PLA itself degrades due to weathering, becoming weaker and developing cracks. Glass fibers, however, are much more stable and help the composite maintain its structural integrity. Even after extensive weathering, GF-PLA retains some mechanical advantages over neat PLA, thanks to the reinforcing effect of the relatively stable glass fibers [53].

Glass fibers often undergo a surface treatment, typically with a silane coupling agent, to improve adhesion with the PLA matrix. This enhances the stress transfer between the fibers and the polymer, leading to better mechanical properties in the final composite. Regarding the GF type and FDM process parameters, the most pronounced effects on the properties of the 3D-printed composites can be summarized as follows:

- The type and orientation of glass fibers in 3D-printed PLA composites significantly influence their mechanical and thermal properties. E-glass fibers are the most common and widely used due to their affordability, good balance of mechanical properties, and compatibility with various resins (including PLA). They offer good tensile strength, stiffness, and dimensional stability when incorporated into PLA filaments. Glass fibers for FDM/FFF applications typically come in diameters ranging from 6 microns to 20 microns. Thinner fibers offer better dispersion within the PLA matrix but may require higher loading percentages to achieve desired strength improvements. Conversely, thicker fibers can provide greater reinforcement but might be more challenging to disperse uniformly and could create printing issues like nozzle clogging.

Continuous glass fibers enhance longitudinal tensile strength but suffer from poor transverse properties due to anisotropic behavior. Short glass fibers (around 250 µm) improve thermal stability, making them ideal for thermally demanding applications. The presence and type of voids formed during 3D printing also play a crucial role in determining the composite’s strength. Comparatively, 3D-printed composites may exhibit reduced mechanical performance compared to injection-molded ones due to differences in fiber orientation [46].

- Influence of Layer Thickness and Orientation: Thinner layers and specific orientations (e.g., flat orientation) improve tensile and flexural strengths. On-edge orientation with +45°/−45° raster angles enhance mechanical strength and modulus [44].
- Extrusion rate and Temperature: Lower extrusion rates and higher printing temperatures improve mechanical properties by enhancing layer adhesion and reducing voids. The impact strength of the composite was found to decrease with increasing extrusion rate, from 2.5 kJ/m^2^ to 1.5 kJ/m^2^ when the extrusion rate was increased from 10 mm/s to 30 mm/s. A higher extrusion rate of 50 mm/s resulted in an impact strength of 1.2 kJ/m^2^ [54,55]. The printing temperature has a significant impact on the mechanical properties of GF PLA composites. A study found that increasing the printing temperature from 190 °C to 230 °C resulted in a significant improvement in tensile strength and modulus of the composite. However, excessive printing temperatures can lead to warping and delamination of the composite. One study found that the printing temperature range of 230 °C to 240 °C resulted in a decrease in tensile strength by 1.02% and a decrease in elastic modulus by 3.32% [56]. Bed temperature also plays a role, with higher bed temperatures (50–70 °C) improving crystallinity and mechanical properties [57].
- Infill Density: The infill density of the composite affects its mechanical properties. A study found that increasing the infill density from 10% to 50% resulted in a significant improvement in tensile strength and modulus of the composite [58]. Another study reported that an infill density of 30% yielded the highest tensile strength and modulus values [59]. A study found that samples with 50% and 75% infill density exhibited higher overall toughness compared to 100% infill density samples [51].

While glass fibers enhance tensile and flexural strength in PLA, they can make the printed parts more brittle, reducing their ability to withstand sudden impacts. Additionally, the abrasive nature of glass fibers can roughen the surface finish and accelerate wear on the printer’s nozzle, requiring more frequent replacements [48].

#### 2.1.3. Plant Fibers

Lignocellulosic fibers are derived from plant biomass (such as stems, leaves, seed husks and so on) and mainly consist of cellulose, hemicellulose, and lignin in various proportions. These natural fibers (NFs) are renewable, biodegradable, and have a low environmental impact. The most used lignocellulosic fibers in conjunction with PLA for FDM 3D printing include, for example, wood pulp fibers, cotton (lyocell), bamboo, sisal, jute, flax, sugarcane bagasse, ramie, henequen and hemp [60], although several other leaf-based materials are also considered (e.g., pineapple leaf fibers, microalgae). Typical fiber loadings used in 3D-printed PLA-based composites usually range from 1 to 10 wt. % [61].

NFs such as hemp, sisal, jute, flax, cotton or kenaf exhibit a range of mechanical and thermal properties that make them suitable for diverse applications. Hemp (Cannabis sativa) boasts high tensile strength (550–900 MPa) and good flexural strength (20–30 MPa), along with low moisture absorption (2–5%) and thermal conductivity (0.03–0.06 W/mK). Similarly, sisal (Agave sisalana) exhibits high tensile strength (400–600 MPa) and comparable thermal properties, including low density (1.3–1.5 g/cm^3^) and high thermal resistance (220–250 °C). Flax (*Linum usitatissimum*) shares these advantageous properties, with tensile strength reaching 500–700 MPa and extremely low moisture absorption (0.5–1.5%). Kenaf (Hibiscus cannabinus) also demonstrates impressive tensile strength (400–600 MPa) and thermal properties, low conductivity and high resistance up to 250 °C. In contrast, jute (Corchorus capsularis) and cotton (Gossypium spp.) exhibit moderate to low mechanical strength, with jute offering a tensile strength of 300–400 MPa and cotton 200–300 MPa, and both also suffer from high moisture absorption (10…20%) and lower thermal stability [62,63,64,65,66].

**Figure 4:**
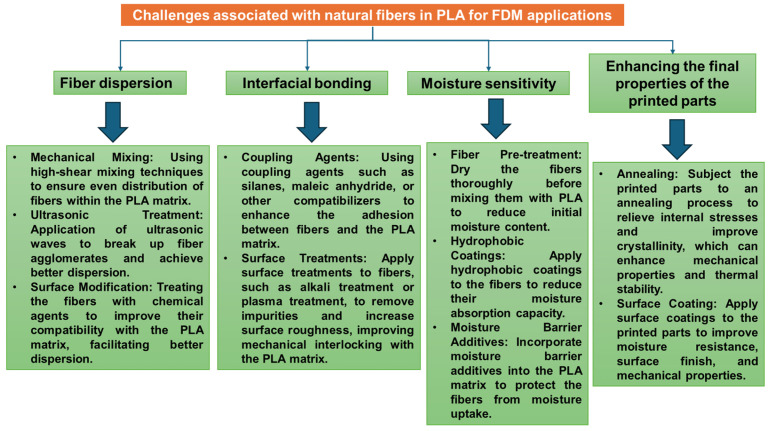
Flow diagram of challenges and mitigation strategies associated with the use of plant fibers in FDM applications.

Lignocellulosic fibers are a natural, renewable, and readily available resource that offers several advantages as a reinforcement material for PLA in FDM 3D printing, such as sustainability, being a biodegradable and eco-friendly alternative to synthetic fibers like glass or carbon fiber, contributing to a more sustainable manufacturing process [67]. They also contribute to the overall composite weight reduction, having a lower density compared to many other reinforcing materials, leading to lighter-weight composites (ranging from 1.1 to 1.2 g/cm^3^, depending on the fiber type and content, neat PLA having an average density of 1.24 g/cm^3^). However, they also present some challenges, such as moisture absorption; lignocellulosics are hygroscopic, meaning they absorb moisture from the environment. The moisture absorption rate can vary from 1% to 15% by weight. This can negatively impact the dimensional stability and mechanical properties of the composite. The properties of the fibers can vary depending on the type of plant, harvesting time, age of the plant, processing methods, and growing conditions. This variability can affect the consistency of the final composite material [68]. Some strategies for mitigation of these shortcomings are presented in the descriptive flow diagram from Figure 4 [69,70].

Inhomogeneous fiber–matrix distribution in natural fiber-reinforced PLA composites for 3D printing remains a major challenge, along with uneven printing and clogging [71]. Plant fibers typically have lower surface energy than synthetic fibers, which can be modified through treatments to improve bonding with PLA. Surface treatments can be applied to lignocellulosic fibers to improve their compatibility with the PLA matrix and enhance the performance of the composite. Common surface treatment methods include [72]:

- Alkali Treatment: This treatment removes impurities, lignin and hemicellulose from the fiber surface, promoting better bonding with the PLA matrix and potentially improving mechanical properties [73].
- Bleaching and pulping: Natural fibers in their raw state harbor various impurities like waxes, oils, and lignin. These substances act as barriers, hindering the formation of strong bonds between the fiber and the PLA matrix. Pulping and bleaching processes effectively remove a significant portion of these impurities, particularly lignin. Lignin, a natural binder in plant cell walls, creates a weak interface with PLA. By eliminating lignin, pulping and bleaching create a cleaner fiber surface, allowing for better adhesion with the PLA matrix. These processes can slightly roughen the surface of the fibers. This increased surface area allows for better mechanical interlocking with the PLA, leading to a stronger bond and improved stress transfer between the fiber and the matrix [74,75]. Pulping and bleaching hemp fibers towards delignified short fibers significantly improves their compatibility with poly(lactic acid) and results in a 52% increase in tensile strength [76].
- Silane Coupling Agents: Silane coupling agents can create a chemical bond between the fibers and the PLA matrix, leading to improved interfacial adhesion and stress transfer, ultimately enhancing the mechanical properties of the composite [77].
- Enzymatic modification: Enzymatic-Assisted Modification of Thermomechanical Pulp Fibers Improves Interfacial Adhesion with Poly(lactic acid) via grafting of less hydrophilic moieties to the lignocellulose fiber surface, e.g., laccase-assisted grafting of octyl gallate (OG) or lauryl gallate (LG) onto the fiber surface [78].
  Studies have shown that the inclusion of NFs in PLA can significantly influence the mechanical, thermal, and physical properties of the resulting composites [77].
- Mechanical Properties: Lignocellulosic fiber reinforcement can improve the tensile strength, modulus, and impact resistance of PLA composites. A study on the tensile behavior of 3D-printed PLA-based composites reinforced with natural henequen fibers found that the tensile strength and modulus increased with increasing fiber loading up to 5 wt. % [32]. Another study on lignocellulose nanofiber/polylactic acid (LCNF/PLA) composites reported that the tensile strength and modulus increased with increasing fiber loading up to 10 wt. % [79]. Research has shown increases in tensile strength by 20–50% and modulus by 30–50% with the incorporation of wood fibers like kenaf and hemp [80]. The addition of natural fibers, such as wood, hemp, or flax, can improve the stiffness and strength of PLA composites. For example, a study found that the addition of 30 wt. % industrial hemp fibers to PLA increased the Young’s modulus by 10.9 Gpa and the tensile strength by 82.9 Mpa [73]. Natural fibers can also improve the toughness of PLA composites by increasing their resistance to crack propagation and improving their impact resistance [81]. The strength of 3D-printed kenaf/PLA composites increases to 3 wt. % but decreases significantly to 5 and 7 wt. % due to voids, and extrusion temperature affects the structure of the filaments [82].
- Wear and friction: NF-reinforced PLA composites exhibit reduced wear rates and friction coefficients, enhancing their suitability for tribological applications. Incorporation of natural fiber mats into the PLA matrix significantly improves wear behavior, resulting in a 10–44% reduction in friction coefficient and over 70% reduction in specific wear rate [83].
- Thermal Properties: The thermal stability of PLA composites with NFs can be slightly affected compared to neat PLA. Some studies report a decrease in the glass transition temperature (T_g_) due to the presence of the fibers [84]. However, the thermal degradation temperature may not be significantly impacted.
- Physical Properties: NF reinforcement can decrease or increase the density of the composite compared to neat PLA, depending on fiber loading and type. Additionally, the hygroscopic nature of these fibers can affect the moisture absorption of the composite, potentially leading to dimensional changes.
- Chemical and moisture stability: PLA composites are more sensitive to high temperatures than to water. The mechanical properties, such as Young’s modulus, decrease significantly at elevated temperatures (80 °C), but the addition of natural fibers helps reduce this decrease [85].

Specifically, the typical mechanical and thermal stability properties of several natural fiber-reinforced PLA printed composites are given in Table 1.

Several fibers’ structural properties and FDM process parameters play a crucial role in achieving good printability and the desired properties in NF-reinforced PLA composites [8,9]:

- Typically, fibers used in 3D printing are short fibers, often between 0.3 and 3 mm in length. Longer fibers can improve tensile strength but may cause issues with printability and nozzle clogging. The diameter of plant fibers can vary widely depending on the type of fiber. Common ranges are from 10 to 100 µm. Also, higher aspect ratio generally leads to better reinforcement. Typical aspect ratios for fibers used in 3D printing are between 50 and 200 [89].
- Printing Temperature: A higher printing temperature can improve the flowability of the PLA matrix and facilitate better fiber dispersion. However, excessively high temperatures can lead to thermal degradation of the wood fibers. The extrusion temperature for PLA-based composites containing lignocellulose fibers in FDM printing generally ranges between 200 °C and 220 °C. This temperature range plays a critical role in influencing the color, physical attributes, and mechanical properties of composites with heat-treated wood fibers, primarily due to changes in the chemical composition of lignocellulosic materials [77]. Research indicates that the PLA matrix in wood–plastic composite (WPC) parts remains amorphous during the FDM printing process, as the crystallization rate of PLA is too slow to achieve full crystallinity [90].
- Infill Pattern and density: The infill pattern can also affect the properties of the composites. A study on the 3D printing of PLA-based composites reinforced with natural lyocell fibers found that the tensile strength and modulus increased with the use of a concentric infill pattern compared to a grid or tri-hexagonal pattern [91]. Infill density significantly influences the improvement of mechanical strength in 3D-printed PLA composites with natural fiber reinforcement. Good tensile strength was observed at 25% of infill for flax fiber-reinforced 3D-printed composites [86]. Optimal load bearing is reported more often at filament crossing angles of −45/+45° [92].
- Build orientation: Generally, NF-PLA composite parts printed with the fibers aligned along the loading direction (e.g., on-edge orientation) exhibit higher flexural strength and modulus compared to parts printed with fibers perpendicular to the loading direction (e.g., flat orientation). The build orientation also influences the surface finish and the need for post-processing steps. For instance, printing a part with a smooth surface finish on the exterior layers can improve the flexural properties by reducing surface defects that could initiate cracks or failures. Additionally, post-processing techniques like annealing or heat treatment can be applied more effectively when the build orientation is considered, further enhancing the flexural properties of the composite part [93].

Also, Mansingh et al. [94] investigated the potential of fabricating fully biodegradable composites using an innovative 3D printing technique, with a particular focus on applications in food and medical product packaging. The weight percentage (wt. %) of powdered raw Pineapple Leaf Fiber (PALF) and alkali-treated PALF in the 3D-printed green composite presented a notable influence on its mechanical and thermal properties. Notably, the incorporation of 3 wt. % alkali-treated PALF yielded the optimal reinforcement, resulting in the highest tensile strength (42.9 MPa) and flexural strength (51.9 MPa). In contrast, composites reinforced with raw PALF exhibited superior ductility, achieving a maximum elongation at break of 6.89%. Additionally, the density of the 3D-printed composites increased proportionally with the wt. % of PALF content. The crystalline structure and chemical bonding characteristics of the composites were characterized through Fourier Transform Infrared (FTIR) spectroscopy and X-ray Diffraction (XRD) analysis. Microstructural analysis further revealed the presence of impurities, voids, and fiber degradation within the 3D-printed composites. These findings underscore the influence of fiber treatment and composition on the performance and structural integrity of the green composites.

Several other studies involving the usage of fibers and PLA for 3D printing applications are summarized in Table 2.

### 2.2. PLA Reinforced with Particles

Some common examples of particle reinforcements for Polylactic Acid (PLA) used in FDM 3D printing, along with their properties and typical loading weights, are as follows:

- Calcium Carbonate (CaCO_3_): This is a widely used mineral filler for PLA. It is inexpensive, abundant, and can improve the stiffness, dimensional stability, and printability of PLA. For example, adding 20–30% by weight of CaCO_3_ particles can increase the modulus and heat deflection temperature of PLA while also improving layer adhesion. A specific product is Omyacarb TF-100, a fine ground calcium carbonate with a median particle size of 1.3 microns, which can be added at 20–30% by weight. A study in the Journal of Polymers and the Environment found that adding 20% CaCO_3_ to PLA increased its flexural modulus by 50% and reduced its thermal expansion coefficient by 30%.
- Carbon Black: Carbon black particles can be used to improve the electrical conductivity and mechanical properties of PLA. They are typically added at a loading weight of 1–10%. For example, a research paper in the Composites Science and Technology journal reported that adding 5% carbon black to PLA increased its electrical conductivity by several orders of magnitude and its tensile strength by 20%.
- Talcum (Magnesium Silicate): Talc is a soft, lamellar mineral that can act as a reinforcing agent and nucleating agent in PLA. It improves stiffness, impact strength, and heat resistance of PLA. For instance, adding 10–20% by weight of talcum particles can enhance the dimensional stability and surface finish of printed parts. An example is Luzenac Talc, which has a median particle size of 1.5 microns and can be added at 10–20% by weight.
- Nanoclay: Nanoclays, such as montmorillonite, are commonly used to improve the barrier and mechanical properties of PLA. They act as nano-scale reinforcements, increasing stiffness, strength, and heat resistance. For example, Cloisite 30B, an organic-modified montmorillonite nanoclay, can be added at 5–10% by weight to PLA to improve its gas barrier properties and mechanical strength. For instance, adding 3% nanoclay to PLA increased its tensile strength by 25% and its flexural modulus by 35% [98].
- Silicon dioxide (SiO_2_): Silicon dioxide particles, also known as silica, can be used to improve the toughness and impact resistance of PLA. They are typically added at a loading weight of 1–5%. For instance, a research paper in the Journal of Applied Polymer Science reported that adding 3% silica to PLA increased its impact strength by 50% and its elongation at break by 100%.
- Metal Powders: Metal particles, such as copper or brass, can be added to PLA to create metallic-looking prints with improved mechanical properties. These particles increase the weight, density, and stiffness of the printed parts. For example, adding 5–15% by weight of brass powder with a particle size of 35–45 microns can create a brass-infused PLA with a metallic sheen.

#### 2.2.1. PLA with Calcium Carbonate

**Figure 5:**
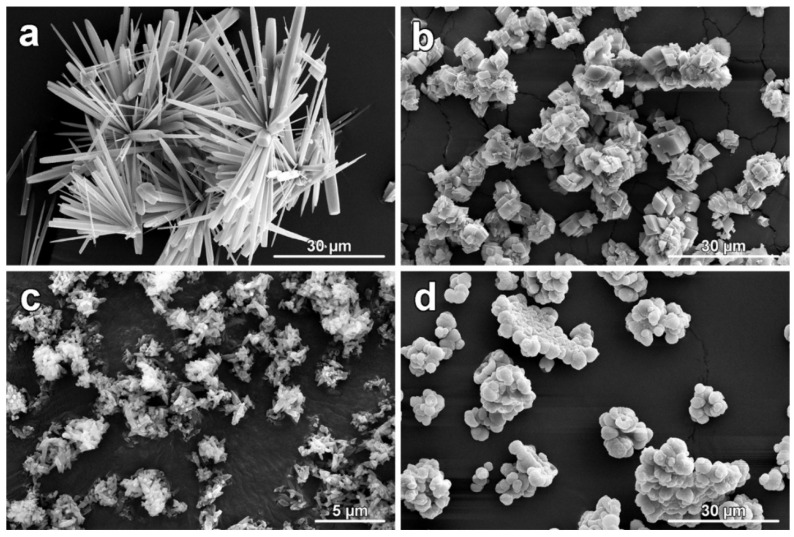
Collection of observed morphologies of synthesized CaCO_3_ polymorphs (aragonite (**a**), calcite (**b**), commercially available calcite (**c**), and vaterite (**d**)) observed under SEM [distributed under the terms and conditions of the Creative Commons Attribution (CC BY) license https://creativecommons.org/licenses/by/4.0/, accessed on 13 June 2024, from [104]].

CaCO_3_ is a naturally occurring mineral that exists in several crystalline forms, including aragonite, calcite, and vaterite [99,100]. These polymorphs have different crystal habits, particle sizes, and surface areas, which can influence the final properties of the PLA filaments [101]. The use of CaCO_3_ particles in PLA filaments can improve their mechanical properties, such as tensile strength and flexural strength, by acting as a reinforcing material [101,102]. Additionally, CaCO_3_ can enhance the thermal stability and resistance to degradation of PLA filaments [101]. Also, this type of composite is researched as a potential biomaterial (ceramic-coated scaffolds), showing potential for further evaluations in bone tissue engineering applications [100]. Regarding the structural and morphological characteristics of calcium carbonate, several parameters influence the properties of the 3D-printed composites [103]:

- Morphology: The morphology of the CaCO_3_ particles, such as spheroidal (granular) or rod-like (Figure 5), can also impact the mechanical properties of PLA filaments. Rod-like particles, for example, have been found to have a more pronounced influence in improving mechanical properties compared to spherical particles. Particles with higher specific surface areas tend to agglomerate more within the PLA matrix, leading to a decrease in mechanical properties [104]. A critical value for the specific surface of calcium carbonate in correlation to PLA was not determined, but as an orientative value, it is around 7 m^2^/g for polyolefin matrices, beyond which increased agglomeration causes a significant decrease in strength and impact resistance [105].
- Amorphous calcium carbonate (ACC) derived from various sources, such as limestone (LS), chalk, or animal origins like white eggshells (WESs), has been explored as a filler material for polylactic acid (PLA) composites. These fillers are particularly beneficial due to their potential to enhance the mechanical properties of PLA-based materials. Notably, the particle size of the ACC plays a significant role in the resulting composite’s performance. Studies have shown that fillers with a particle size of 32 μm tend to exhibit superior tensile strength compared to those with a larger particle size of 63 μm. This suggests that smaller particles contribute more effectively to the reinforcement of the PLA matrix, likely due to their higher surface area, which facilitates better interaction with the polymer matrix. In addition to tensile strength, the tensile modulus of PLA composites is significantly influenced by the filler content. For amorphous calcium carbonate-based fillers, the tensile modulus increases as the filler content rises, indicating enhanced stiffness and structural integrity. The highest tensile modulus was observed at a filler content of 20 wt. % for both 32 μm and 63 μm particle sizes, suggesting that this concentration represents an optimal balance between filler loading and the material’s mechanical properties. When comparing the toughness of different ACC sources in PLA composites, it was found that limestone (LS)-based fillers provided superior toughness compared to those derived from white eggshells (WESs). This difference in toughness is likely due to variations in the microstructure and the intrinsic properties of the fillers, which influence the energy absorption capacity of the composite materials. Overall, the use of amorphous calcium carbonate fillers, particularly from limestone, shows promising potential in improving the mechanical performance of PLA composites, offering a viable option for enhancing the material’s strength and durability for various applications [106].

The processing parameters for printing PLA-CaCO_3_ composites, referenced from the literature, are as follows [107,108,109]:

- Nozzle Temperature Optimal Range: 190 °C to 210 °C. Higher temperatures can improve the dispersion of CaCO_3_ particles within the PLA matrix, improving the mechanical properties.
- Printing Speed: 50 to 80 mm/s. Slower speeds can lead to better particle dispersion and improved mechanical properties.
- Infill Density: 50% to 80%. Higher densities can enhance the mechanical properties of the printed part, especially with higher CaCO_3_ loads.
- Infill Pattern: Use a grid or honeycomb pattern for better stress distribution and to take advantage of the improved stiffness of PLA-CaCO_3_ composites.
- Layer Height: 0.1 to 0.3 mm. Fine layers can improve the surface finish and mechanical properties, especially with high-quality prints.

**Figure 6:**
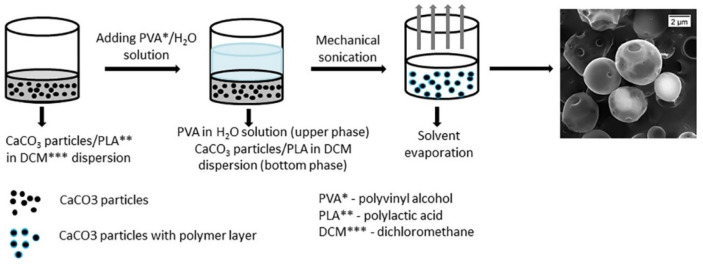
Fabrication of PLA CaCO_3_ hybrid micro-particles [reproduced with permission from [111].

A low-permeability polylactic acid (PLA) coating to calcium carbonate (CaCO_3_) micro-particles was successfully applied, which encapsulated co-precipitated bovine serum albumin-fluorescein isothiocyanate (BSA–FITC), utilizing the solvent/oil/water (S/O/W) emulsion technique (Figure 6) [110,111]. This effectively sealed the micro-particle pores and significantly decreased the shell permeability. The incorporation of an additional polyvinyl alcohol (PVA) layer significantly improved the stability of the coated microparticles and refined the size distribution of the resultant PLA/CaCO_3_ particles. The formulation of the particles was optimized with a PVA concentration of 2.5%, which was found to be the most effective for achieving a stable and uniform particle size distribution. To gain a deeper understanding of particle formation, the varying mass ratios of CaCO_3_ microparticles to PLA were systematically examined across different samples. Bovine serum albumin-fluorescein isothiocyanate (BSA–FITC) was observed within the PLA particles across all formulations, with the CaCO_3_/PLA mass ratio ranging from 0.1 to 1.2. At CaCO_3_/PLA mass ratios below 0.8, the polymer-coated microparticles exhibited increased resistance to ethylenediaminetetraacetic acid (EDTA) treatment, thereby maintaining the integrity of their bioactive cargo. In contrast, at a CaCO_3_/PLA mass ratio of 1.2, the PLA-coated particles displayed increased susceptibility to EDTA due to the thinner PLA coating on each CaCO_3_ particle and the presence of pores in the PLA shell. This compromised the stability of the bioactive content within the particles. Based on these findings, a CaCO_3_/PLA mass ratio of 0.8 was determined to be optimal, as it provided the highest protein payload while maintaining the stability of the microparticles against dissolution. This ratio offers a favorable balance between bioactive content retention and the structural integrity of the particle coating.

Both PLA and CaCO_3_ are biodegradable materials [112], making them highly suitable for biomedical applications. Given that CaCO_3_ is a natural component of bone, this microcapsule system holds potential for the storage and controlled delivery of bone growth factors or other pharmaceutical agents aimed at treating bone-related diseases. Additionally, the system could provide enhanced mechanical support during cargo delivery. Advanced delivery strategies, such as targeted drug delivery, could be realized by incorporating magnetic nanoparticles into the PLA shell. This modification would enable magnetic navigation, allowing precise delivery of drug-loaded microcapsules to specific target sites. The PLA-coated CaCO_3_ microparticles, encapsulating bioactive molecules, are anticipated to have diverse applications in biomedicine, particularly as efficient drug storage and delivery systems. Furthermore, the hybrid microcapsules developed in this study could be employed for the encapsulation of a wide range of water-soluble substances, including proteins, insulin, cytostatics, and polysaccharides. This versatility underscores the potential of these microcapsules as a multifunctional platform for various therapeutic and pharmaceutical applications

#### 2.2.2. PLA with Zinc Oxide

Polylactic Acid-Zinc Oxide (PLA-ZnO) nanocomposites have emerged as versatile and multifunctional materials, suitable for a wide array of applications due to their exceptional antibacterial, photocatalytic, and ultraviolet (UV) absorption properties. Their superior antibacterial performance offers a significant advantage over pure polymers by reducing the risk of infection through fomite transmission. Despite these promising attributes, the current utilization of PLA-ZnO nanocomposites, predominantly produced via conventional fabrication methods, remains limited to simple geometries such as films and fibers. To date, few studies have explored the additive manufacturing (AM) of PLA-ZnO nanocomposites, primarily due to challenges in material processing and the production of filaments with properties that can be reliably preserved post-3D printing [113].

As research advances to overcome these challenges, the integration of PLA-ZnO nanocomposites into AM has the potential to expand their application spectrum significantly. AM offers a distinct advantage in fabricating complex geometries and customized components, which could unlock novel uses for these nanocomposites. This intersection of 3D printing with advanced antibacterial materials is garnering increasing interest within the biomedical field, with the goal of creating personalized antibacterial medical devices tailored to individual patient anatomies. Furthermore, this innovation aligns with the priorities of the post-pandemic AM industry, aiming to deliver safer and more hygienic materials for enhanced environmental security. Nevertheless, a critical concern is whether the functional properties of PLA-ZnO nanocomposites, particularly their antibacterial effectiveness, can be retained at sufficient levels after undergoing the AM process. The transition from conventional manufacturing to AM introduces additional processing steps, such as high-temperature extrusion and deposition, which may alter the intrinsic properties of the nanocomposite materials. Understanding the impact of these processes on the structural integrity and functionality of PLA-ZnO nanocomposites remains a key research focus, as it will determine the feasibility of their broader implementation in additive manufacturing [114].

#### 2.2.3. PLA with Silicon Carbide and Graphite

Silicon carbide (SiC) and graphite are chosen as fillers for polylactic acid (PLA), primarily due to their mechanical and thermal attributes. A significant reduction in recovery time is observed for composites with high filler loading. For instance, a composite comprising 50 wt. % carbon and 10 wt. % SiC in a PLA matrix exhibits an 87% decrease in recovery time compared to pure PLA [115]. The evaluation of shape recovery performance is based on three key parameters: recovery ratio, recovery rate, and recovery time. The recovery ratio is defined as the proportion of the initial deformation angle restored after the recovery process. The recovery rate represents the instantaneous time derivative of the recovery ratio, providing insight into the speed of shape restoration. Recovery time is measured as the interval between the initiation of the thermal trigger and the point at which the recovery rate reaches its maximum value. A detailed methodology for the extrusion of printer filaments and the subsequent 3D printing process is thoroughly described to ensure reproducibility and clarity.

By altering the material composition, and consequently its thermal conductivity, the rate of shape memory response can be effectively controlled. The correlation between material composition and shape recovery rate, determined through thermal conductivity analysis, offers a framework for designing structures capable of activating shape memory responses at tailored rates. The advanced capabilities of 3D printing, particularly the use of multiple filaments and print heads, enable the fabrication of structures with highly heterogeneous material compositions. This approach represents a significant departure from conventional methods, such as variations in temperature or structural thickness, that have traditionally been used to modulate the timing of shape memory response activation within structures.

**Figure 7:**
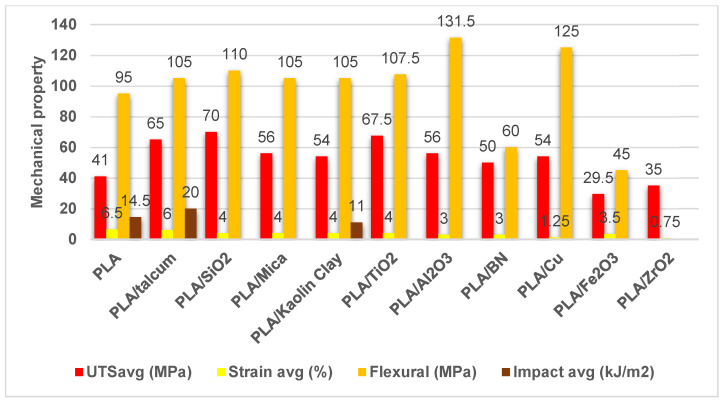
Average values for the mechanical properties of 3D-printed PLA reinforced with inorganic additives: neat PLA [116]; talcum [117,118]; silica (SiO_2_) [119,120]; mica [121,122]; kaolin clay [123]; titania (TiO_2_) [124,125]; alumina (Al_2_O_3_) [126]; boron nitride (BN) [127]; copper (Cu) [128]; iron (III) oxide (Fe_2_O_3_) [129]; zirconia (ZrO_2_) [130].

Several mechanical properties (UTS: ultimate tensile strength (MPa); strain at break (%); flexural strength (MPa) and impact strength (kJ/m^2^)) of 3D-printed PLA reinforced with various inorganic fillers selected from the literature are given in Figure 7. In this figure, the average value was calculated from the samples sets presented in the indicated studies.

Overall, the inclusion of various fillers, such as, notably, titanium dioxide (PLATiO_2_) and copper (PLA/Cu), appears to improve the mechanical properties of the PLA material compared to the baseline PLA without any fillers. 

### 2.3. PLA Reinforced with Carbon-Based Nanomaterials

The most effective nanomaterials for enhancing the mechanical properties of PLA in FFF/FDM 3D printing are [131,132,133]:

- Carbon nanotubes (CNTs): Several studies have shown that incorporating CNTs into PLA can significantly improve its tensile strength, modulus, and toughness, while reducing elongation at break. The mechanical properties are influenced by interfacial adhesion and CNT dispersion.
- Graphene nanoplatelets (GNPs): Adding GNPs to PLA can enhance its mechanical properties, depending on factors such as raster direction and nanoplatelet size. Larger GNPs and optimizing raster orientation can improve the mechanical capabilities of graphene-reinforced PLA.
- Short carbon fibers (SCFs): Incorporating SCFs into PLA has been found to enhance its tensile and shear modulus in various printing directions. Continuous carbon fibers can also increase the tensile and flexural behavior of PLA, although they may reduce failure strain.
- Combinations of carbon nanofillers: Using multiple carbon nanofillers, such as GNPs and multi-wall carbon nanotubes (MWCNTs), can produce PLA nanocomposites with better mechanical properties compared to single fillers. For example, adding SCFs and graphene to PLA tripled its mechanical strength [134].

The maximum filler content is typically limited to avoid drastically decreasing the elastic strain of the composites and to allow adequate extrusion of the filaments during FFF/FDM printing. Optimizing printing parameters, such as temperature and infill, can also influence the mechanical properties of PLA nanocomposites.

The addition of carbon nanotubes to polylactic acid (PLA) composites during FDM printing can significantly improve the mechanical, thermal, and electrical properties of the resulting materials. CNTs act as reinforcing agents, increasing the mechanical strength and stiffness of the PLA matrix by providing additional load-bearing capacity and resistance to deformation. This aspect can improve the dimensional accuracy of 3D-printed PLA parts by reducing the warpage and shrinkage that can occur during the printing process. The strong interfacial bonding between CNTs and PLA matrix enhances the mechanical properties of the composite by reducing the likelihood of debonding and improving the transfer of stresses between the CNTs and the matrix. These nanofillers can form a network structure within the PLA matrix, which enhances the mechanical properties of the composite by providing additional mechanical reinforcement and improving the resistance to deformation [135,136].

The CNT content has a significant influence on the PLA/CNT composite, with the optimal CNT loading depending on the specific application and desired properties. The mechanical properties improve by increasing CNT content up to a certain threshold, beyond which the benefits may plateau or even diminish due to issues like CNT agglomeration [137]. Studies comparing CNTs with other reinforcements like cellulose nanocrystals (CNCs) also show that CNTs provide superior improvements in mechanical properties [138].

- Effect of CNT Content on Thermal Properties: The addition of CNTs can improve the thermal conductivity of the PLA matrix, which can lead to improved mechanical properties by reducing thermal gradients and stress concentrations within the composite. The presence of CNTs can accelerate the crystallization of PLA, leading to improved mechanical properties by increasing the degree of crystallinity and reducing the likelihood of defects and imperfections within the matrix. The presence of CNTs and other nanofillers like nanoclay increases the thermal stability and storage modulus of PLA composites, making them more robust at higher temperatures [139].
- Effect of CNT Content on Electrical Properties: The electrical conductivity of PLA/CNT composites has also been investigated, with the addition of CNTs being shown to significantly improve the electrical conductivity of the materials. The CNT content has a significant influence on the electrical properties of PLA/CNT composites during FDM printing. The electrical resistivity varied from approximately 1 × 10^12^ Ω/m^2^ to 1 × 10^2^ Ω/m^2^ for CNT contents ranging from 0 wt. % to 8 wt. % [139].
- Effect of CNT on the mechanical properties: The optimal carbon nanotube (CNT) content for achieving the best mechanical properties in PLA/CNT composites is still a topic of ongoing research. However, studies have shown that a CNT content of around 5–10 wt. % can result in significant improvements in mechanical properties (10–60% in tensile strength, up to 30% for flexural strength) compared to neat PLA [140]. It has been found that a 6% CNT content led to a substantial increase in tensile and flexural strength, as well as improved electrical conductivity [139]. However, in other studies, it has been found that while CNT inclusion increased the Young’s modulus by 30% at 5% CNT loading, it reduced tensile strength and overall toughness [137].

CNT structure, as well as the FDM process parameters such as extrusion temperature, building direction, infill percentage and geometry, and layer height were key factors in determining the properties of the printed materials [33].

- CNT structure and dimensions: The type (single-walled carbon nanotubes, SWCNTs, multi-walled carbon nanotubes, MWCNTs) and length of CNTs play crucial roles in determining the performance of 3D-printed parts. Longer carbon nanotubes (typically 0.65–1.3 mm) result in higher thermal and electrical conductivities, but no appreciable change in the mechanical properties of PLA/CNT composites during FDM printing [141]. SWCNTs have a more significant impact on the material properties than MWCNTs, but they are generally more expensive. Long MWCNT inclusion determines better performance at lower concentrations [142].
- Nozzle temperature: The optimal printing temperature range for PLA-CNT composites is between 180 °C and 220 °C, with an optimal printing temperature of around 200 °C for tensile strength and 220 °C for flexural strength. A higher printing temperature can lead to improved bonding between the PLA matrix and CNTs, resulting in improved mechanical properties, but may also reduce the impact strength of the composite material [143]. Higher extrusion temperatures can reduce the void fraction in the printed parts but may also result in less alignment of CNTs due to radial flow and fusion between adjacent layers [137]. For better electrical and thermal conductivity, a lower temperature of 200 °C is preferred [139].
- Layer height and infill geometry: The specimens printed at 0.100 mm layer height, gyroid-type infill geometry and number of perimeters of 6 have maximum tensile strength [144]. For better thermal and electrical conductivity, greater layer heights are more desirable [139].
- Build direction: The mechanical properties of 3D-printed PLA composites exhibit significant anisotropy based on build orientation. Tensile strength and Young’s modulus are higher when the material is printed in a flat orientation compared to an upright orientation [93].

Functionalized MWCNTs can significantly enhance the mechanical properties of polymer composites. The functionalization of carbon nanotubes (CNTs) through mild acid treatment enhances their nucleating effect during polylactic acid (PLA) crystallization and promotes better dispersion of the CNTs within the PLA matrix. The PLA/f-CNT nanocomposite exhibits higher degrees of crystallinity, as expected, because f-CNTs act more efficiently as nucleating agents than c-CNTs.

**Figure 8:**
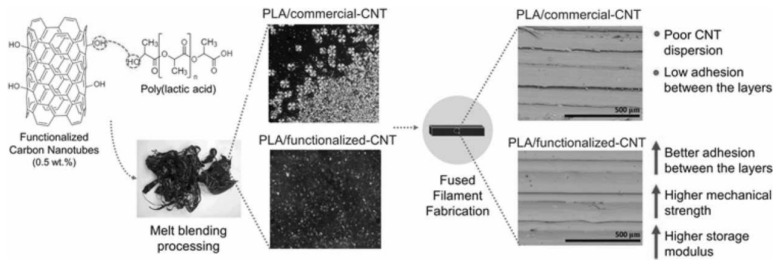
Differences between PLA/commercial-CNT and PLA/functionalized-CNT [reproduced with permission from [145].

Differential Scanning Calorimetry (DSC) analysis revealed no thermal property gradients in 3D-printed specimens across all analyzed samples, including pure PLA, PLA/c-CNT, and PLA/f-CNT. This uniformity in thermal properties, encompassing transition temperatures and degrees of crystallinity, was consistent across the top, central, and base layers of the printed specimens. Furthermore, the 3D printing process did not alter the melting temperature of the materials, indicating that the thermal stability of the composites remained unaffected. The surface functionalization of carbon nanotubes (CNTs) significantly improved the composite’s mechanical performance. Specifically, the PLA/f-CNT samples exhibited a 43% higher storage modulus at body temperature (37 °C) compared to PLA/c-CNT samples, indicating that functionalized CNTs act as more effective reinforcing agents. Scanning Electron Microscopy (SEM) imaging of the fracture interfaces further revealed enhanced interlayer adhesion in PLA/f-CNT samples (Figure 8). This improvement is attributed to the better dispersion of functionalized CNTs within the PLA matrix, resulting in a more uniform material flow during the printing process. Under tensile stress, PLA/f-CNT samples demonstrated markedly superior mechanical properties, with layer-to-layer adhesion contributing to a significant increase in tensile strength (from 29.4 ± 0.7 MPa in PLA/c-CNT to 41.6 ± 1.4 MPa in PLA/f-CNT). These findings highlight the efficacy of CNT surface modification in enhancing the thermal and mechanical properties of 3D-printed PLA/CNT nanocomposites. In particular, the study underscores the role of mild acid treatment (HNO_3_, 5 mol L^−1^) in functionalizing CNTs and optimizing their reinforcing potential [145].

### 2.4. Natural and Synthetic Polymer Blends with Biomass Fillers

#### 2.4.1. Hydroxypropyl-Methylcellulose-Reinforced PLA

Biocompatible hydroxypropyl methylcellulose (HPMC)/polylactic acid (PLA) composites were successfully fabricated as filaments and 3D-printed parts using fused deposition modeling (FDM). This study examined the effects of HPMC content on various properties, including microstructure, chemical structure, thermal behavior, mechanical performance, and water contact angle (CA). The results demonstrated a uniform distribution and dispersion of HPMC within the PLA matrix. Incorporating HPMC up to a concentration of 7% did not affect the chemical structure of PLA during the melt blending and filament extrusion processes. However, HPMC incorporation significantly influenced the thermal properties of the composites, leading to an increased glass transition temperature and a reduced cold crystallization temperature [146]. While the tensile strengths of the composite filaments were comparable to those of neat PLA, the tensile and impact strengths of the 3D-printed samples decreased as the HPMC content increased. This reduction was attributed to increased porosity induced by the presence of HPMC. Additionally, the incorporation of HPMC resulted in a lower water contact angle, which indicates enhanced hydrophilicity. This characteristic could be particularly advantageous for applications in the biomedical field, where surface wettability plays a critical role.

#### 2.4.2. PLA with Lignin and Polymerized-Lignin-Treated NFC

Composite PLA filaments containing unmodified and lignin/polymerized lignin surface-modified nanofibrillated cellulose (NFC) were analyzed to assess their mechanical, thermal, and structural properties. The addition of NFC was found to reduce the stretchability, toughness, breaking tenacity, bending stiffness, and compression resistance of the filaments, while slightly enhancing their initial modulus. Despite a marginal toughening effect imparted by NFC, the improvement was limited due to poor interfacial interactions between NFC and the PLA matrix [147].

The mechanical performance of the composite filaments was closely associated with the NFC’s surface modification and concentration within the PLA matrix. When the NFC content was increased to 5 wt. %, the mechanical properties showed the most significant deterioration. However, incorporating lignin/polymerized lignin surface-modified NFC mitigated the decline in mechanical properties and resulted in an increase in storage modulus. The observed limitations in toughness improvement were attributed to poor interfacial adhesion and the presence of NFC agglomerates. These agglomerates, characterized by irregular plate-like shapes and uneven dispersion, acted as structural weak points, thereby impairing the mechanical integrity of the filaments. Scanning Electron Microscopy (SEM) images revealed the structural inconsistencies, while Fourier Transform Infrared (FTIR) analysis confirmed interactions between lignin-modified NFC and the PLA matrix. Dynamic Mechanical Analysis (DMA) further demonstrated a notable increase in elasticity with the addition of modified NFC. Although no significant differences in thermal stability were observed between filaments containing unmodified and modified NFC, the composite filaments exhibited an enhanced crystallization rate compared to neat PLA [148].

Despite only minor variations in mechanical properties, the incorporation of NFC improved the thermal stability of the composites. Lignin, known for its antioxidant properties and UV protection capability, enhanced the thermal oxidative resistance of the filaments, thereby extending the lifespan of 3D-printed objects. Additionally, the light-brown and dark-brown hues imparted by lignin/polymerized lignin surface-modified NFC conferred a unique and aesthetically appealing appearance to the filaments and their corresponding 3D-printed constructs [149].

#### 2.4.3. PLA with Acetylated Tannin

This study investigated the properties and 3D printability of a composite material composed of Polylactic Acid (PLA) and Acetylated Tannin (AT). The acetylation of tannin enhanced its dispersion within the PLA matrix, enabling the successful fabrication of PLA/AT composite filaments using a twin-screw extrusion process. The inclusion of 20 wt. % AT in the PLA matrix did not significantly compromise the tensile properties of the composite. Thermogravimetric analysis indicated that the PLA/AT composites could be safely processed at 3D printing temperatures below 250 °C to avoid thermal decomposition. While acetylated tannin improved the overall printability of PLA systems, optimal printability was achieved at temperatures below 220 °C to prevent phase separation and aggregation of AT, especially at higher loading levels [150].

The incorporation of acetylated tannin at varying concentrations did not substantially affect the melting and glass transition temperatures of PLA, as it had minimal impact on the intermolecular interactions or chain flexibility of the PLA polymer. However, a reduction in the crystallinity of PLA/AT composites was observed, which accelerated their degradation in aquatic environments, particularly under alkaline conditions. This accelerated degradation could be advantageous for short-term biomedical applications, such as implantable devices, where controlled degradation is a desirable attribute [151].

#### 2.4.4. PLA with Cork

Biodegradable composites were synthesized, composed of cork and Polylactic Acid (PLA) with varying proportions of cork. A decline was observed in the tensile mechanical attributes of the composites as the proportion of cork was augmented. The impact resistance demonstrated an initial decrease with the incorporation of cork; however, it exhibited an increase with further escalation in the cork content. The viscoelastic characteristics of the composites displayed a diminishing trend with the increase in cork content. In contrast, the specific modulus and specific strength of the composites increased with higher cork content, highlighting the potential of cork–PLA composites for lightweight and impact-resistant 3D-printed structures. This improvement in specific mechanical properties underscores their suitability for advanced engineering applications. A filament composed of a 5% *w*/*w* cork–PLA composite was successfully developed, demonstrating compatibility with fused deposition modeling (FDM) processes. The 3D-printed cork–PLA composite exhibited slightly reduced tensile mechanical properties compared to its compression-molded counterpart. However, an exception was observed in elongation at break, where the 3D-printed composite displayed superior ductility, emphasizing its potential for applications requiring enhanced flexibility and resilience [152].

#### 2.4.5. PLA/Wood Flour

Composite filaments of Wood Fiber (WF) and Polylactic Acid (PLA) were synthesized, and their properties were evaluated in this study. Additionally, 3D specimens were fabricated utilizing the Fused Deposition Modelling (FDM) technique. The study tells us that WF/PLA composite filament demonstrated compatibility with the FDM process, indicating its suitability for 3D printing. Moreover, the incorporation of WF resulted in alterations in the microstructure of the PLA fracture surface. The interfaces between the WF and PLA were distinctly visible and the resistance to initial deformation of the composite was observed to be enhanced upon the addition of WF, in comparison to pure PLA. Also, the onset temperature for thermal degradation of the composites exhibited a slight decrease, while the final residual ratio post-thermal decomposition of the composites showed an increase and the addition of WF at a concentration of 5 wt. % did not influence the melting temperature of the PLA. [153]

Parameters of 0.2 mm layer height, 0.7 mm nozzle diameter, 75% fill density, and 35 mm/s velocity maximize the flexural strength of wood–PLA composite parts built through FFF. [154]

#### 2.4.6. PLA/HA with PCL Addition

The fabrication of bespoke materials, composed of biocompatible and biodegradable polymers either independently or in conjunction with mineral components, resulted in printable substances possessing chemical stability and mechanical attributes conducive to bone regeneration. The mechanical characteristics were validated to meet the mechanical threshold requisite for trabecular bone applications. The tailoring of degradation rates exhibited a strong correlation with the material composition, indicating an enhancement through the integration of mineral phases, such as hydroxyapatite, which further accelerated the degradation of PLA–PCL amalgamations. This, in conjunction with the introduction of regulated porosity and scaffold architecture, could potentially facilitate the control and equilibrium of biomaterial resorption and neo-osteogenesis as required [155].

#### 2.4.7. PLA with TPS and ESO

This research is driven by the current market demand for cost-effective alternatives to Polylactic Acid (PLA). In this study, novel compositions based on PLA, modified by Thermoplastic Starch (TPS) and Epoxidized Soybean Oil (ESO), were developed to enhance the ductility of PLA and reduce the cost of the products without compromising their biodegradability [156]. The efficacy of the proposed compositions was validated by studying their rheological, mechanical, and thermal properties, water resistance, and compostability. An increase in the Melt Flow Rate (MFR) value resulted in enhanced adaptability to injection and blow molding processes, thereby expanding the range of short-life application products from a technological perspective. To evaluate the suitability of the obtained PLA/TPS (thermoplastic starch) blends for various packaging industry applications, their mechanical properties were thoroughly analyzed. The incorporation of epoxidized soybean oil (ESO) resulted in materials with enhanced softness, improved impact strength (up to 16.69 kJ/m^2^), and increased tensile and ductile properties, including an elongation at break of approximately 8.8%, compared to native PLA. Scanning Electron Microscopy (SEM) of fractured surfaces confirmed these improvements. Additionally, the presence of ESO in the blends was observed to delay water diffusion into the matrix, thereby potentially improving the dimensional stability of products subjected to short-term water exposure. The modified TPS, prepared with ESO, was shown to be processable using standard PLA machinery, facilitating its integration into existing production systems. Furthermore, the ability to substitute up to 25% of PLA with ESO-modified TPS offers a cost-effective alternative while maintaining comparable properties and compostability to that of pure PLA, underscoring its potential as a sustainable material for packaging applications [157].

Several other studies involving the usage of different fillers and PLA for 3D printing applications are summarized in Table 3.

## 3. Conclusions

In the dynamic landscape of additive manufacturing, the quest for high-performance materials continues, and our exploration of reinforced PLA composites has revealed promising innovations and practical applications. The integration of natural fibers into PLA enhances strength and stiffness, offering an eco-friendly alternative while maintaining mechanical integrity. By incorporating metallic particles, researchers have pushed the boundaries of PLA’s capabilities, improving thermal and electrical properties and expanding their utility in functional parts. PLA composites reinforced with cellulose fibers demonstrate excellent printability and mechanical performance, with the synergy between PLA and cellulose opening doors to sustainable materials for various industries. Carbon fibers provide significant improvements in tensile strength, sometimes exceeding a 100% increase when loaded at 15%, though these materials require careful handling to prevent clogging and ensure uniform dispersion. Glass fibers enhance flexural strength and thermal stability but tend to make composites brittle, reducing impact resistance. Natural fibers, such as kenaf and hemp, improve sustainability and weight reduction but are limited by moisture absorption and mechanical stability, which can affect layer adhesion. Among particle reinforcements, calcium carbonate improves stiffness, dimensional stability, and printability, though large particles can cause agglomeration and reduce mechanical performance if not properly managed. Carbon nanotubes (CNTs) show potential in improving mechanical, thermal, and electrical properties, though their effectiveness depends on proper dispersion and bonding within the PLA, with surface treatments enhancing their effects. However, challenges like clogging, dispersion issues, and moisture absorption remain common for both synthetic and natural fibers in FDM printing, requiring adjustments in nozzle size, temperature, and other parameters to facilitate smoother flow. Continuous carbon fiber composites stand out for high-strength applications but require specialized equipment and processing, while natural fibers align with eco-friendly goals but underperform compared with synthetic fibers like carbon and glass, which offer superior properties. Among particle-based systems, calcium carbonate is particularly promising for improving mechanical properties and printability without significant processing challenges. Suitable printing parameters, including higher temperatures (230–250 °C) for particles, lower temperatures (below 220 °C) for natural fibers, increased infill density (50–70 %), slower printing speeds (10–20 mm/s), and lower layer heights (0.1–0.2 mm), are critical for maximizing performance, particularly in fiber-reinforced composites. While each reinforcement system presents unique benefits and challenges, continuous carbon fibers and calcium carbonate-based composites appear to offer the most promising results depending on the application. The optimization of printing parameters, such as nozzle size, temperature, layer height, and infill density, is crucial for achieving the best outcomes. Reinforced PLA composites align with global efforts to reduce reliance on fossil-based polymers, and the journey toward optimized PLA composites involves collaboration across disciplines, including materials science, engineering, and design. By bridging the gap between theory and practice, reinforced PLA composites hold immense promise for a greener, more resilient additive manufacturing industry.

## Figures and Tables

**Table 1:** Comparison of mechanical properties of PLA composites reinforced with different types of natural lignocellulose fibers [data compiled from [86,87,88]] *.

Fiber Type	Tensile Strength (MPa)	Tensile Modulus (Gpa)	Flexural Strength (MPa)	Impact Resistance	Thermal Stability
Flax	50	5	60	High	Good
Jute	40	3	40	Medium	Medium
Sisal	30	2	30	Low	Low
Kenaf	45	4	50	Medium	Medium
Ramie	35	3	35	Low	Low
Cotton (lyocell)	25	2	25	Low	Low

* For comparison’s sake, the values corresponding to the mechanical properties were chosen as the median value for the respective composite type at the closest to 5 wt. % fiber loading.

**Table 2:** Composite filaments and 3D-printed materials involving PLA and fibers.

Reinforcement/Additive	Description	Properties	Applications	Reference
Cellulose nanofiber (CNF)	The CNF/PLA compounds were obtained by blending a PEG surface-modified CNF aqueous suspension with microscale PLA powder, followed by air-drying using various mass ratios of CNF to PLA, 0.25:99.75, 0.5:99.5, and 1:99, respectively.	Tensile strength 50.7 MPa, Young modulus E, 3.73 MPa	Environmentally friendly method which significantly reduced the use of chemical reagents and shortened the processing time.	[95]
steam-exploded coconut fiber (F) and PLA or PLA/PBS	PBS content: 0 or 20 wt. %; F content: 3, 6 or 10 wt. %	Impact strengths of PLA97/F3 and PLA77/PBS20/F3 were approximately 8.5% and 7.4% higher than those of the original PLA and PLA/PBS, respectively. During the degradation test, both PLA97/F3 and PLA77/PBS20/F3 exhibited higher tensile strengths than the original materials.		[96]
short lyocell fibers	Lyocell fibers (FCP400) with a nominal length of 400 µm, loading reported to PLA: 10%, 20%, 30%	By combining fiber fibrillation, matrix modification, and post-printing annealing, we achieved an excellent balance of tensile strength (85 MPa), Young’s modulus (7.2 GPa), and strain at break (3.2%)—the highest reported values for such composites.	performance structures using 100% bio-derived materials	[91]
Lignocellulose nanofiber/polylactic acid (LCNF/PLA) composite	Cellulose raw material used was hardwood bleached kraft pulp (HBKP) with a degree of polymerization (DPv) of ~750; cooking active alkali charges of 10%, 13%, 16%, and 19% (calculated by sodium oxide) with a liquor-to-chip ratio of 1:4.	The flexural strength of the CNF/PLA composite increased from 92.7 MPa to 151.2 MPa by combining 10% CNF (without lignin) with PLA. The flexural strength of LCNF/PLA composite with internal lignin content of 3.7% (0.37% of the total mass) was increased from 151.2 MPa to 234.5 MPa, which is 153.0% higher than that of pure PLA.		[79]
Carbon nanotubes (CNTs) were coated on short glass fibers (SGFs)	CNT-coated SGFs through a one-step flame synthesis technique. SGF- and CNT-SGF-reinforced composite filaments were fabricated with filler loadings of 1, 5 and 10 wt. %	Specimens using SGF- (≥5 wt. %) and CNT-SGF (1–5 wt. %)-reinforced PLA filaments exhibited higher Young’s modulus and tensile strength values due to the enhanced interface adhesion. Furthermore, the FDM printing raster angles (±45° and 0°/90°) did not noticeably affect the tensile properties of the samples made of the same material.		[97]

**Table 3:** Composite filaments and 3D-printed materials involving PLA and various natural and synthetic fillers.

Type	Reinforcement/Aditive	Description	Properties	Applications	Reference
Particle and fiber hybrid-reinforced PLA	MXene (Ti_3_C_2_T_x_) and recycled carbon fiber (rCF)	MrCF (fiber mass fraction 10 wt. % with 1 wt. % Ti_3_C_2_T_x_) and 178 g of dried PLA	Compared to pure PLA, the modified PLA displayed significant improvements: 15.6% in toughness, 112.1% in flexural strength, and 31.8% in notched impact strength.	The modified PLA demonstrated superior electromagnetic shielding performance due to the absorption properties of the composite material.	[158]
Particle-based composite	Ferronickel slag (FNS)	composites with FNS contents of 2.0, 6.0, 10.0, and 14.0 wt. %	PLA/5.0 wt. % FNS composite exhibited the most significant improvement in mechanical properties, with a roughly 18% increase in both tensile and flexural strength compared to unfilled PLA thermoplastic		[159]
	Magnesium	1% Mg reported to PLA	Among the 3D-printed samples with different infill orientations (θ = 0°, 45°, ±45°, 90°), the highest UTS was measured in specimens with 0° infill orientation (UTS = 43 MPa), whereas the lowest UTS was measured for the 90° infill orientation (UTS = 26 MPa). Mg particles also reduce the mechanical properties at all infill orientations.		[160]
	Parawood (*Hevea brasiliensis*)	Four different parawood powder weight ratios were blended with PLA, encompassing 0%, 5%, 10%, and 15% (*w*/*w*)	The maximum tensile strength was attained when the parawood powder content was 15% *w*/*w*.	Large-scale screw-extrusion 3D printing process for producing furniture parts	[161]
	Pyrolyzed HSC (hazelnut shell carbon)	2 % HSC was added to the PLA matrix	The 3D-printed PLA-HSC parts demonstrated excellent photothermal performance with a light absorption intensity of around 93%	Low-cost, compostable, high-efficiency photothermal conversion materials with shaping freedom	[162]
	Salix alba sawdust	1%, 2%, 3%, and 4% (weight %) Salix alba were loaded in the PLA matrix.		PLA-salix alba-based composite feedstock has the controlled MFI and may be used for scaffold preparation in biomedical applications.	[163]
	PLA/Olive wood waste	olive wood scraps (with an amount ranging between 10 and 20% by weight)	The use of wooden scraps in place of virgin PLA granulate reveals a rise in the impact in the extrusion (ca. 27%) and 3D printing (11%) stages	Environmental benefits resulting from the inclusion of wood scraps in PLA filaments (20% of wood corresponds to 10% environmental impact reduction) supporting further research in this area.	[164]
	Cu_2_O nanoparticles (50-100 nm diameter)	PLA-3 wt. % Cu_2_O mixture	Superior mechanical performance of the 3D-printed nanocomposite at a 0° raster angle, while the mechanical properties gradually decreased for raster angles of 45° and 90°.	This composite can be utilized for the fabrication of various prostheses featuring specific groove geometries, bio parts in medical devices, robotics surgical systems, and implants	[165]
	PLA/MgAl_2_O_4_:Sm^3+^ composite filaments	Composite filaments of PLA with different amounts of phosphors (0, 1, 2, 3 and 4 phr)	Luminescence intensity of the composites regularly increased with the inorganic phosphor contents	Luminescent bioplastic composite filaments. Filaments and samples of the composites emitted pink-orange light under UV light	[166]
Sheet composite	graphene oxide (GO)	different content of GO (0.4, 0.8, 1.6, 2.4 and 4.0 wt. %) reported to PLA	PLA/GO scrolled fibers and printed fibers with graphene content of 0.4 wt. % exhibit outstanding mechanical properties with strength increases of about 32.7% and 35.2%.		[167]
Polymer-based composite	PLA-TPU	50%, 70% and 90% TPU reported to PLA	The UTS values for PLA50, PLA70, and PLA90 were achieved at 27.27 MPa, 40.91 MPa, and 54.18 MPa, respectively. Also, the fracture toughness results were consistent with the mechanical properties. The PLA90 had 1.69 and 2.36 times higher fracture toughness than the PLA70 and PLA50 compounds, respectively.	By increasing the amount of TPU, the printability decreased due to higher melt strength and viscosity, incomplete melting, feeding problems caused by buckling, and incomplete integration between adjacent rasters and layers.	[168]
	thermoplastic starch (TPS)/poly(lactic acid) (PLA)/poly(butyleneadipate-co-terephthalate) (PBAT) composite	Ratio of TPS:PLA:PBAT was fixed at 50:40:10 wt. %.	113% increase in elongation at break and the 190% rise in impact strength compared to PLA	highly renewable filaments for 3D printing	[169]
	PLA with biodegradable elastomer poly (butyleneadipate-co-terephthalate) (PBAT) and poly (methyl methacrylate) (PMMA)		Tensile strength and breaking elongation were 94.8% and 3650%, respectively, of that of 3D-printed pure SC-PLA	Controlled compatibilization effect of PMMA endows PBAT with good dispersibility in the quaternary system, without affecting the hierarchical crystallization of enantiomeric PLA matrices for complete stereo-complexation	[170]
	Chitosan	PLA and 10 wt. % chitosan	The acidic environment caused by the degradation of PLA can be counteracted by chitosan, probably due to its protonation.	Biocomposite materials	[171]

## Abbreviations

PLA	polylactic acid
FDM	fused deposition modeling
ABS	poly (acrylonitrile-co-butadine-co-styrene)
PETG	polyethylene-terephthalate glycol
FFF	fused filament fabrication
PHB	polyhydroxy butyrate
PBS	polybutylene succinate
CPA-PLA	carbon fiber-reinforced polylactic acid
GFs	glass fiber
OG	octylgallate
LG	lauryl gallate
PALF	pineapple leaf fiber
FTIR	Fourier transform infrared
XRD	X-ray diffraction
HBICP	hardwood bleached kraft pulp
LCNF	lignocellose nanofiber
SGFs	Short glass fibers
LS	limestone
WES	white eggshell
BSA-FITC	Bovine serum albumin -fluorescein isothiocyanate
S/O/W	solvent/oil/water
DCM	dichloro methane
UTS	Ultimate tensile strength
BN	Boron nitride
SCFs	short carbon fibers
MWCNTs	Multiwalled carbon nanotubes
SEM	scanning electron microscopy
AT	acetylated tannin
DMA	dynamic mechanical analysis
PCL	polycaprolactone
ESO	Epoxidized soybean oil
TPS	Thermoplastic starch
MFR	Melt flow rate
FNS	Ferronickel slab
PBAT	polybutylene adipate-co-terephthalate
PMMA	poly(methyl methacrylate)
f-CNTs	functionalized carbon nanotubes
phr	parts per hundred resin (polymer)
